# Posttranscriptional Regulation of Insulin Family Ligands and Receptors

**DOI:** 10.3390/ijms140919202

**Published:** 2013-09-18

**Authors:** Amaresh C. Panda, Ioannis Grammatikakis, Je-Hyun Yoon, Kotb Abdelmohsen

**Affiliations:** Laboratory of Genetics, National Institute on Aging-Intramural Research Program, National Institutes of Health, Baltimore, MD 21224, USA; E-Mails: amaresh.panda@nih.gov (A.C.P.); yannis.grammatikakis@nih.gov (I.G.); je-hyun.yoon@nih.gov (J.-H.Y.)

**Keywords:** glucose homeostasis, insulin-like growth factor, insulin-like growth factor receptor, RNA-binding protein, micro RNA, long noncoding RNA, mRNA decay, mRNA translation, insulin signaling, alternative splicing IRES

## Abstract

Insulin system including ligands (insulin and IGFs) and their shared receptors (IR and IGFR) are critical regulators of insulin signaling and glucose homeostasis. Altered insulin system is associated with major pathological conditions like diabetes and cancer. The mRNAs encoding for these ligands and their receptors are posttranscriptionally controlled by three major groups of regulators; (i) alternative splicing regulatory factors; (ii) turnover and translation regulator RNA-binding proteins (TTR-RBPs); and (iii) non-coding RNAs including miRNAs and long non-coding RNAs (lncRNAs). In this review, we discuss the influence of these regulators on alternative splicing, mRNA stability and translation. Due to the pathological impacts of insulin system, we also discussed the possibilities of discovering new potential regulators which will improve understanding of insulin system and associated diseases.

## 1. Introduction

Diabetes is one of the most common diseases in the United States accounting for about 25 million individuals and the number is expected to reach 40 million by 2050 [1]. Diabetes is also linked to a high risk of certain cancers such as breast, colon, breast, prostate, and bladder cancer [2,3]. Disruption of insulin system is the main reason for the disease development. Glucose directly enhances insulin secretion and synthesis in pancreatic β-cells in the initial stages without affecting transcription [4]. This response is usually quick and efficient in healthy individuals but can be altered due to genetic or acquired factors causing impairment in glucose metabolism and high susceptibility to develop diabetes. Insulin and insulin-like growth factors (IGFs) share structural similarities and common signal pathways named “insulin and IGF signaling” (IIS). These ligands also share common receptors including insulin receptors (IR) and insulin-like growth factor receptors (IGFR) [5].

Insulin system including insulin, IR, IGF, and IGFR is regulated at the posttranscriptional levels by the *trans-*acting factors of splicing regulatory RNA-binding proteins (RBPs), turnover and translation regulator RNA-binding proteins (TTR-RBPs), and non-coding RNAs including microRNAs (miRNAs) and long non-coding RNAs (lncRNAs). These factors mainly regulate pre-mRNA alternative splicing and mRNA stability and translation. Upon transcription, pre-mRNA is processed, exons are joined and introns are removed by a complex of ribonucleoproteins called spliceosome [6,7]. About 90% of the human genes which contain more than one exon undergo alternative splicing to generate multiple mRNA variants from a single pre-mRNA [8,9]. Alternative splicing is a major factor that contributes to protein isoform diversity [10]. Splicing regulatory RNA binding proteins bind to the pre-mRNA and promote or suppress spliceosome formation on the alternative splice sites [7]. Splice variants are well characterized in the insulin system, however not all alternative splicing factors are known. For example while *IR* pre-mRNA splicing is regulated by factors such as CELF and MBNL [11,12], regulators of insulin pre-mRNA splicing are not known. TTR-RBPs are also involved in the regulation of insulin system including PTBP which regulates the stability of *insulin* mRNAs [13,14]. Other TTR-RBPs, summarized in this review, such as HuR and hnRNP C regulate the translation of *IGF-1R* mRNA while Lin-28 and IMPs regulate *IGF-2* mRNA translation [15–17]. Additionally, recent RNA-sequencing analysis identified other RBPs associated with pre-mRNA and mRNAs encoding for proteins in the insulin system suggesting that they could have additional posttranscriptional roles in glucose homeostasis.

Non-coding RNAs like miRNAs and lncRNAs also play key roles in posttranscriptional regulation of gene expression [18–20]. While miRNAs typically bind mRNA and negatively regulate stability or translation, lncRNAs are diverse in their effects. lncRNAs are involved in splicing, mRNA stability and translation, and also act as decoy for miRNAs or RBPs as reviewed previously [20]. miRNAs RNAs are involved in the regulation of insulin system. For instance let-7f, miR-1, miR-125b, and miR-100 regulate the expression of IR, IGF-1, IGF-2, and IGF-1R respectively [21–24]. Although lncRNAs are not yet directly involved in posttranscriptional regulation of insulin system, HI-LNC25 affects pancreatic β-cells development; H19 regulates IGF-1R through miR-675-3p, while Airn was recently reported to regulate *IGF-2R* transcription [25–27].

In this review, we will focus on posttranscriptional regulation of insulin, IGFs and their receptors (IR and IGFR) by splicing regulatory RNA binding proteins, TTR-RBPs, and non-coding RNAs.

## 2. Posttranscriptional Regulation of *Insulin* mRNA

Insulin is synthesized and secreted in response to glucose treatments by a marked increase in mRNA translation within 1 h. This quick release is accompanied by fast and efficient splicing and translation of insulin transcripts [4]. Transcribed mRNAs are subjected to *trans-*acting factors including RBPs and non-coding RNAs such as miRNAs and lncRNAs [18,28].

### 2.1. Insulin Alternative Splicing

The human insulin gene contains three exons and the 5′ untranslated region (5′ UTR) covers exon 1 and a part of exon 2 which harbors the translation initiation site. This gene encodes for proinsulin (insulin precursor), which is further processed to insulin upon proteolytic cleavage. In pancreatic islets intron 1 was found to contain a cryptic splice site that extends exon 1 by 26 bases generating a longer 5′ UTR. This splice variant is translated by four to six-fold more efficiently due to altered secondary structure of the 5′ UTR which may facilitate ribosome accessibility on the site [29]. In addition, this splice variant was found to be metabolically regulated and its expression increases with high levels of glucose [29]. Intron 1 retention due to a single nucleotide polymorphism in human insulin was found to generate a longer 5′ UTR and enhance *insulin* mRNA translation efficiency [30]. Similarly, intron 1 retention in mouse *insulin 2* mRNA alters the secondary structure of the 5′ UTR and increases mRNA translation efficiency without affecting its stability [31]. Another variant of the mouse *insulin 2* mRNA containing 12 bases deletion on exon 2 has higher translation efficiency most likely due to a loss or gain of interaction with unidentified *trans-*factors such as RBPs, miRNAs or lncRNAs [32].

Although the alternative splicing regulators are not known, these findings suggest a tight regulation of *insulin* pre-mRNA alternative splicing, particularly in intron 1, generating multiple mRNA variants with alterations in translation efficiency.

### 2.2. Regulation of Insulin Expression TTR-RBPs

TTR-RBPs are group of RBPs which regulate mRNA stability and translation. *Insulin* mRNA is relatively short and thus few RBPs are known to bind and influence either stability or translation. Several studies revealed the presence of a conserved stem-loop structure in the 5′ UTR [33,34]. Knight and Docherty showed that a number of proteins can bind to the insulin 5′ UTR and hypothesized that these proteins may control insulin expression [34]. The impact of RBPs on insulin expression is discussed below.

#### 2.2.1. PABP

The poly(A)-binding protein (PABP) contains four RNA recognition motifs (RRMs) [35]. Normally PABP binds the 3′ poly(A) tail of eukaryotic mRNA which is essential for poly(A) shortening and translation initiation [36]. It is also capable of binding 5′ UTR to regulate key steps in mRNA translation [37,38]. PABP was found to bind *insulin* mRNA both at the 5′ and 3′ UTRs enhancing translation. The binding of PABP to the 5′ UTR of *insulin* mRNA was recently found to be regulated by protein disulphide isomerase (PDI). This enzyme interacts with PABP and alters the disulphide bonds leading to increased binding of PABP to *insulin* 5′ UTR and eventually leads to higher insulin translation during high glucose condition [39].

#### 2.2.2. HuD

This RBP belongs to the ELAV (embryonic lethal abnormal vision)/Hu (human) group of proteins which comprises HuR, HuB, HuC, and HuD. Elav/Hu proteins are known to bind U- and AU-rich RNA sequences of target transcripts through three highly conserved RRMs and implicated in the regulation of stability and translation [40,41]. HuD is mostly expressed in neuronal cells but recently it has been shown to be present in other tissues such as liver, testis and pancreatic β-cells [42,43]. In pancreatic β-cells HuD, but not HuR, was found to bind a 22 nt-long segment in the 5′ UTR of *insulin* mRNA immediately upstream of the translation start site. *Insulin* mRNA translation analysis revealed that HuD specifically suppressed translation through binding to the 5′ UTR. Glucose treatment induced dissociation of HuD from *insulin* mRNA leading to increased translation [43]. These findings indicate that although HuD is not abundant in pancreatic β-cells compared to neuronal cells, it has a negative impact on insulin production. However, it is not clear if HuD is posttranslationally modified with glucose treatment. It is also not known whether other Hu proteins such as HuB or HuC are expressed in pancreatic β-cells and if they are involved in insulin regulation.

#### 2.2.3. PTBP

Polypyrimidine tract-binding protein (PTBP) is a member of the heterogeneous nuclear ribonucleoprotein family which was first found to bind polypyrimidine-rich sequence [44]. This RBP contains four RRMs through which it binds short pyrimidine-rich sequences and long pyrimidine tract containing cytosine. PTBP has been implicated in several cellular processes such as splicing, polyadenylation, mRNA stability and translation initiation [45]. PTBP binds the polypyrimidine-rich sequence in the 3′ UTR of rat *insulin* mRNA to promote mRNA stability [46]. This binding is enhanced by glucose treatment upon which PTBP was found to enhance the stability of *insulin* mRNA [13,46]. Fred and colleagues observed that PTBP specifically binds to the 5′ UTR of *insulin* mRNA *in vitro*. They suggested that binding of PTBP to an internal ribosomal entry site (IRES) in the 5′ UTR of *insulin* mRNA enhances mRNA translation by 40%–100% in human β-cells in a cap- and eIF4A-independent mechanism during starvation and stress to uphold the basal insulin synthesis [47].

Together, we hypothesize that basal *insulin* mRNA is under translation repression by HuD through binding to the 5′ UTR. This repression is relieved by glucose treatment which stimulates the dissociation of HuD from *insulin* mRNA likely due to protein modification such as phosphorylation. Then simultaneously PDI is stimulated to modify PABP to enhance its binding to *insulin* mRNA leading to increased mRNA stability. Also, PTBP increases the half-life of *insulin* mRNA to enhance insulin biosynthesis.

## 3. Posttranscriptional Regulation of Insulin Receptor

Glucose homeostasis in mammals depends on insulin secretion by pancreatic β-cells and the IR which mediates cellular response and signaling events upon binding to the secreted insulin [48]. Insulin receptor is a hetero-tetramer transmembrane glycoprotein with two extracellular α-subunits where insulin binds and two membrane-spanning β-subunits. Both α- and β-subunit are encoded by the same gene and are separated with proteolytic cleavage [49]. Insulin binding to IR leads to a conformational change resulting in *trans-*phosphorylation of the intracellular β-subunits and tyrosine phosphorylation of other substrates [50–52]. Normally, insulin binding to IR activates phosphatidylinositol 3-kinase (PI3K)–protein kinase (Akt) pathway and disruption of which can lead to diabetes [53]. Like *insulin* mRNA, *IR* mRNA is also regulated posttranscriptionally by alternative splicing events, TTR-RBPs, and non-coding RNAs.

### 3.1. RBPs in Alternative Splicing of Insulin Receptor

The gene encoding IR consists of 22 exons of which the 36-nt long exon 11 is alternatively spliced resulting in two isoforms; IR-A (lacks exon 11) and IR-B (includes exon 11). The 36-nt encodes 12 amino acids within the intracellular segment of IR-B α-subunit [54,55]. The IR-A isoform has a higher binding affinity for insulin as well as faster internalization and recycling time [56,57]. The IR-B isoform is the predominant isoform expressed in insulin-sensitive tissues like skeletal muscle, liver and kidney [54,55,58]. Exon 11 skipping occurs in a number of abnormalities and disease states such as type II diabetes, myotonic dystrophy, obesity, cancer, and aging [59–61]. Splicing of *IR* pre-mRNA is regulated by splicing regulators including CELF, hnRNPs, MBNL, SR proteins, Staufen1 and RBM4 as discussed below.

### 3.2. CELF

CELF (CUG-binding protein and Elav-like family member) are RNA binding proteins that bind and regulate mRNA alternative splicing, editing, and translation [12,62]. CELF1 (formerly known as CUGBP1) is the first discovered regulator of IR exon 11 splicing. It promotes exclusion of exon 11 through binding to two sites, one upstream of exon 11 and one in the middle of exon 11 itself [63].

### 3.3. hnRNPs

The heterogeneous nuclear ribonucleoprotein (hnRNP) family proteins are involved in several processes including pre-mRNA splicing, mRNA export, stability and translation [64,65]. Elevated levels of hnRNP H in myotonic dystrophy type 1 (DM1) inhibit IR exon 11 splicing [66,67]. hnRNP H also interacts with CELF1 in an RNA-dependent manner leading to maximum inhibition of IR exon 11 inclusion in normal myoblasts. hnRNP A1 and hnRNP F also regulate alternative splicing of exon 11. They bind AGGGA sequences in intron 10 and have opposite effects on IR exon 11 splicing. While hnRNP F enhances, hnRNP A1 inhibits exon 11 inclusion and both effects are eliminated by the deletion of the GA-rich elements [68].

### 3.4. SR Proteins

SR proteins are alternative splicing regulatory proteins that contain Serine-Arginine domains for protein–protein interactions and they usually regulate alternative splicing by promoting exon inclusion. They also play roles in mRNA export and translation [69]. SRSF1 (serine/arginine-rich splicing factor 1, formerly known as ASF1/SF2) competes for binding to the splice site with hnRNP A1 and a point mutation (U→C) at the −3 position decreases hnRNP A1 and increases SRSF1 binding to abolish the effect of hnRNP A1 on IR splicing [68]. Also, SRSF1 along with SRp20 recognizes exon splicing enhancers in exon 11 and promote exon inclusion [63].

### 3.5. MBNL

Muscleblind-like (MBNL) proteins have been implicated in the regulation of alternative splicing of pre-mRNAs [10,66]. MBNL1 is the first splicing factor reported to bind and positively regulate *IR* pre-mRNA exon 11 inclusion in muscle cells [11]. It has been proposed that it directly binds to a cluster of binding sites downstream of exon 11 [70,71]. MBNL proteins antagonize the action of CELF proteins in several alternative splicing events including IR exon 11 [66,72]. In addition, MBNL1 was found to interact with other splicing regulators such as hnRNP H involved in the regulation of *IR* pre-mRNA splicing as explained above [66,67]. In normal myoblasts increased levels of MBNL1 lowers the inhibitory effects of hnRNP H on IR splicing [66].

### 3.6. Staufen 1

Staufen 1 (Stau1) is a double stranded RNA binding protein involved in mRNA transport, localization, and, decay [73–75]. Stau1 was recently found to be highly expressed in DM1 skeletal muscle and identified as a regulator of splicing in myogenic C2C12 cells through direct interaction with *IR* pre-mRNA [73]. However, it is not clear if Stau1 exerts its regulatory effect through binding to a specific sequence (exon 11 for example) within *IR* pre-mRNA. It is also not known if Stau1 interacts with other splicing factors to regulate IR splicing.

### 3.7. RBM4

The RNA-Binding Motif Protein 4 (RBM4) binds RNA with two recognition motifs and regulates pre-mRNA alternative splicing and translation [76,77]. RBM4 null mice display low insulin levels, smaller pancreatic islets, and impaired glucose tolerance suggesting that RBM4 is involved in the regulation of insulin expression [76]. Indeed, RBM4 overexpression enhanced *insulin* mRNA expression and promoted exon 11 inclusion in *IR* pre-mRNA in glucose stimulated pancreatic acinar AR42J cells [76]. RBM4 was found to preferentially bind to a GC-rich motif *in vitro*. However, it is not known if RBM4 binds GC-rich sequences in *IR* pre-mRNA or mature mRNA which may synergistically function with IR splicing regulators.

Together, these finding improved our understanding of *IR* alternative splicing. Although SR proteins have been proposed to regulate alternative splicing of exon 11, the mechanism is not clear. It is possible that they antagonize with CELF1 either by recruiting the spliceosome on exon 11 or by blocking CELF1 binding [63]. Also, CELF and MBNL have been shown to target similar alternative splicing events with opposite outcomes on the mature transcripts, during development and myotonic dystrophy [11,72]. Similarly, MBNL1 and hnRNP H have also been shown to differentially regulate IR exon 11. Although the mechanism is not clear, RNA-independent interaction may play a role in this process [66]. It is possible that a combination of the above regulators determines the outcome of exon 11 splicing and the expression of IR-A and IR-B isoforms. The abundance or the post-translational modifications of the splicing regulators may be critical for *IR* exon 11 alternative splicing depending on the physiological state of the cell. Future studies will shed more light on a combined mechanism through which these factors govern *IR* mRNA alternative splicing.

### 3.8. Regulation of Insulin Receptor Expression by TTR-RBPs and miRNAs

mRNAs are usually subjected to posttranscriptional regulation by RBPs and non-coding RNAs mostly through binding to 3′ UTR of target mRNAs and regulate their translation and/or stability. *IR* mRNA translation has been shown to be regulated in a cap-independent manner with the presence of IRES in the 5′ UTR [78]. Although several splicing factors are known to regulate alternative splicing of *IR* pre-mRNA, only PTBP1 and PTBP2 are known to regulate *IR* mRNA translation. They bind CCU motif upstream of the *IR* translation start site in 5′ UTR which enhances IRES-mediated *IR* mRNA translation in brain cells [79].

miRNAs are small non-coding RNAs that regulate gene expression at the posttranscriptional levels. They interact with target mRNAs with partial complementarity forming miRNA-induced silencing complex (RISC) to suppress translation or promote degradation [18]. They are involved in many cellular processes and pathological conditions including cell survival, proliferation, differentiation, cancer, and diabetes [23,80–82]. Insulin sensitivity and glucose homeostasis are controlled by the let-7 miRNA family [21]. Overexpression of let-7 reduced insulin secretion and impaired glucose tolerance in mice. They regulate the abundance of IR and IR substrate 2 suggesting potential pathological and clinical relevance of let-7 in type 2 diabetes [21,83].

## 4. Posttranscriptional Regulation of IGF and IGFR

Insulin-like growth factor (IGF) and IGF receptor (IGFR) are key factors in IGF signaling axis [84]. This pathway leads to the activation of PI3K to promote cell growth, proliferation, differentiation, survival and migration [85]. Unbalanced levels of IGF cause the appearance of pathological conditions such as diabetes, cancer and premature aging [86]. It has been reported that mice with lower IGF live 40% longer and resist disease [87]. Like insulin and IR, IGF and IGFR are regulated by alternative splicing, RPBs and non-coding RNAs. In this section we review these regulators and their impact on the expression of IGFR and its ligand IGFs.

### 4.1. IGF and IGFR Alternative Splicing

IGF-1 is initially translated as a pro-IGF-1 protein that is proteolytically processed to give rise to mature IGF-1 and E peptide from the *C*-terminal end of the precursor protein. *IGF-1* gene consists of 6 exons that undergo alternative splicing generating different mRNA variants [88]. Exons 3 and 4 are constitutively spliced in the mature transcript and they encode for the IGF-1 protein. Exons 5 and 6 are alternatively spliced giving rise to different E peptides [89,90]. In humans, there are 3 splice variants of IGF-1. Variant one includes exon 5 (*IGF-1Ea*), variant two includes exon 6 but not exon 5 (*IGF-1Eb*), and variant three includes a part of exon 5 (49 bases) along with exon 6 (*IGF-1Ec*). *IGF-1Ea* and *IGF-1Eb* were recently found to be essential for myogenic differentiation [91]. IGF-1Ea splice variant induces muscle hypertrophy in transgenic mice and enhances myopathic phenotypes [92,93]. IGF-1Ec, also known as mechano-growth factor (MGF), serves as a local tissue repair since it is highly expressed in muscle, bone and tendon following damage resulting from mechanical stimuli and in brain and heart following ischemia [94]. It has been suggested that the splice variant MGF enhances the number of progenitor cells in human normal and disease muscle cell cultures [95]. This may facilitate repair of post-mitotic tissue through satellite cell activation, proliferation and fusion [96]. Additionally, exons 1 and 2 contain two alternative transcription start sites that generate transcripts with different 5′ UTRs [97]. Longer 5′ UTR in rat IGF-1 was found to be associated with less mRNA translation efficiency [98]. Recently, an additional IGF-1 splice variant lacking exon four was identified in a large kindred with familial short stature with a single nucleotide mutation on the donor splice site of intron 4. This variant is suggested to be associated with mental retardation; however, it remains to be determined if it causes the disease or the symptoms associated with it [99].

The 30 kb long *IGF-2* gene contains 9 exons, but only exons 7, 8 and part of exon 9 are translated to encode the protein. There are different transcripts of IGF-2 due to different promoter usage (P0–P4) that are active depending on the developmental stage [100–102]. Transcripts undergo alternative splicing of the first 6 exons depending on the promoter [101]. Although the coding region always remains the same, alternative splicing leads to a great variability of the 5′ UTR of the transcripts and subsequently differential translation or stability regulation as discussed in this review.

Although different splice variants are well characterized, the splicing factors that regulate processing of IGF-1 and IGF-2 are largely unknown. The alternative splicing regulator SRSF1 was found to bind a purine-rich sequence (18-nt long) in exon 5 of IGF-1 to enhance its inclusion in the mature transcript [103]. As mentioned above, inclusion of exon 5 is found in IGF-1Ea, which is associated with myogenic differentiation, muscle hypertrophy, and myopathic phenotypes [91–93]. Exon 5 inclusion was also increased in muscle biopsies obtained from patients treated with growth hormones [103,104].

The IGF-1 Receptor shares numerous structural and functional similarities with the Insulin Receptor gene. The amino acid sequence homology is more than 50% and the *IGF-1R* gene size resembles the size of IR. Also, the number and size of exons shows remarkable homology but IGFR lacks an exon similar to exon 11 in *IR* pre-mRNA [105]. The two known isoforms of *IGF-1R* differ by three nucleotides, resulting in a change in the sequence of the transmembrane domain of the β subunit [106]. These two *IGF-1R* isoforms show similar binding affinity to IGF-1 but the shorter isoform shows higher receptor stimulation and signal transduction capacity and reduced receptor internalization rates [107]. Thus far, no splicing factor has been identified as regulator of these alternative splicing events of *IGF-1R* pre-mRNA.

### 4.2. Regulation of IGF and IGF-1R by TTR-RBPs

In recent years many TTR-RBPs have been shown to regulate the stability and translation of *IGFs* and *IGFR* mRNAs. In this part we focus on the effects of TTR-RBPs on the stability and translation of mRNAs encoding for IGF and IGFR.

#### 4.2.1. Nocturnin

Nocturnin is a circadian deadenylase which is ubiquitously expressed with high abundance in liver, kidney, and testis [108]. Nocturnin is localized in the cytoplasm where it binds mRNAs and is involved in the regulation of mRNA decay as reviewed previously [109]. Interestingly, Nocturnin deficient mice remain lean on high fat diet suggesting that it may play a role in lipid metabolism [110]. Nocturnin was found to bind the long 3′ UTR of *IGF-1* mRNA, which contains potential regulatory motifs involved in mRNA degradation to suppress the expression of IGF-1. Overexpression of Nocturnin lowered the levels of *IGF-1* mRNA suggesting that binding enhances mRNA degradation in mice [108].

#### 4.2.2. HuR

The RBP HuR is a member of the Elav/Hu proteins as explained above. HuR mostly binds the 3′ UTR of several mRNAs to regulate the stability or translation [15,111]. It is thus involved in many cellular processes such as proliferation, cell survival, apoptosis and pathological conditions like cancer [40,112,113]. In some cases HuR also binds the 5′ UTR and interferes with IRES mediated mRNA translation. For example, HuR binds *thrombomodulin* RNA and suppress its IRES mediated translation in IL-1 beta treated lung adenoma A549 cell line [114]. Similarly, HuR also binds the 5′ UTR of *IGF-1R* transcript and suppress its translation. It has been suggested that this is mediated by the dual effects of HuR; delaying cap-dependent translation initiation and blocking IRES mediated translation initiation [15]. Additionally, amino acid deprivation enhanced HuR binding to *IGF-1R* mRNA and reduced IRES activity, while mitotic block by nocodazole lowered HuR binding and enhanced IRES activity in cancer cells [115]. Stress conditions are known to alter HuR binding to its target mRNAs. For example, HuR complex with *SIRT1* mRNA is dissociated by oxidative stress due to singling events that stimulated HuR phosphorylation by the CHK2 [116]. In fact, amino acid starvation increases cytoplasmic HuR concentration [117]. Thus, stress such as amino acid deprivation or oxidative stress may alter HuR localization or phosphorylation which could alter the binding to *IGF-1R* mRNA interfering with translation initiation. However, these possible mechanisms remain to be investigated. Also, it appears that Hu proteins, HuR and HuD are negative regulators of *insulin* and *IGF-1R* mRNA translation through binding to the 5′ UTR implicating that both Hu proteins might synergistically regulate insulin levels and insulin signaling.

#### 4.2.3. hnRNP C

hnRNP C is a member of the heterogeneous nuclear ribonucleoprotein (hnRNP) family proteins. As explained above hnRNP A1, hnRNP F, and hnRNP H are involved in *IR* pre-mRNA splicing. hnRNP C is also involved in pre-mRNA processing and interacts with AU-rich sequences in 5′ UTR and 3′ UTR of several mRNAs to regulate translation and stability [64,65]. For example, hnRNP C was shown to bind the 3′ UTRs of *urokinase receptor* mRNA promoting its stability [64]. It also binds the 3′ UTR of *amyloid precursor protein* (*APP*) mRNA and promotes mRNA translation [65]. While HuR binds the 5′ UTR of *IGF-1R* mRNA and suppresses translation, hnRNPC competes with HuR for IRES binding to promote IRES-mediated mRNA translation in human breast tumor cells [115]. Under stress conditions such as amino acid deprivation, HuR shuttles to the cytoplasm, competes with hnRNP C, and suppresses *IGF-1R* mRNA translation. Since hnRNP C also undergoes posttranslational modifications, it may be phosphorylated under stress conditions, an event that may stimulate dissociation from *IGF-1R* mRNA allowing HuR to suppress mRNA translation.

#### 4.2.4. PTBP

As reviewed above, PTBP binds polypyrimidine-rich sequences, regulates the stability of rat *insulin* mRNA, and is suggested to bind the 5′ UTR of *insulin* mRNA to regulate IRES-mediated translation [44,118]. The 5′ UTR of *IGF-1R* mRNA contains an IRES sequence that binds PTBP to allow cap-independent mRNA translation in rat vascular smooth muscle cell and human MCF-7 cells [119]. Overall, PTBP synergistically regulate insulin and IGF-1R expression through binding to the IRES of those mRNAs to promote translation.

#### 4.2.5. Lin-28

Lin-28 is a highly conserved RNA-binding protein which is involved in RNA processing, particularly miRNAs [120]. It suppresses let-7 biogenesis through binding to let-7 precursor, facilitating its terminal uridylation and degradation [120]. In C2C12 mouse myoblasts, Lin-28 was found to bind the *IGF-2* and *myod1* mRNA to enhance translation [16]. However, the target site of lin-28 in the 5′ UTR of *IGF-2* and its role in IRES-mediated mRNA translation has not been elucidated. This regulatory effect could be essential for muscle cell differentiation since Lin-28 expression is induced in differentiated muscle cells which enhance the expression of IGF-2 [16]. Through its influence on miRNA biogenesis, particularly let-7, Lin-28 can also indirectly regulate IGFR expression in the insulin system as discussed below.

#### 4.2.6. IMPs

*IGF-2* mRNA binding proteins (IMPs) is a family of three closely related proteins (IMP1, IMP2, and IMP3) which bind *IGF-2* mRNA and implicated in susceptibility to type 2 diabetes. They contain two RRMs and four heterogeneous nuclear ribonucleoprotein K-homology (KH) domains [121]. IMPs bind target mRNAs and regulate stability, localization, and translation [122]. *IGF-2* gene generates 5 transcripts with different 5′ UTRs but identical coding regions and 3′ UTRs from 5 different promoters (P0–P4) [101,123]. These mRNAs are called *IGF-2 leader 1* (*IGF-2 L1*) to *IGF-2 L4* mRNAs. *IGF-2 L2* and *IGF-2 L3* mRNAs contain IRES in the 5′ UTR that mediates translation initiation [123]. *IGF-2 L3* mRNA contains IMPs binding site while *IGF-2 L4* mRNA is constitutively translated and does not have binding site for IMPs [124]. However, it has been suggested that IMP3 interacts with *IGF-2 L3* and *IGF-2 L4* mRNAs to enhance mRNA translation in K562 cells without affecting their stability [122]. Additionally, overexpression of IMP3 in mouse melanoma cells B16F10 increases tumor growth and metastasis by increasing IGF-2 expression [125]. It is also reported that IGF-2 abundance in different tumors correlates with IMP3 levels without changes in *IGF-2* mRNA suggesting that IMP3 is a translational activator [122]. Binding of IMP2 to the IRES of IGF-2 L3 was found to be regulated by the mammalian target of rapamycin (mTOR) pathway. mTOR interacts with and phosphorylates IMP2 in two sites (Ser162/164) and this double phosphorylation increases its binding to *IGF-2 L3* mRNA and subsequently promote translation through a cap-independent IRES mechanism [17]. This dual phosphorylation of IMP2 is found in mouse embryo and is likely to enhance the expression of IGF-2 to support fetal growth. While phospho-IMP2 is ubiquitously expressed in adult tissues it appears more abundant in the pancreatic islets of langerhans [17]. This is likely to promote IGF-2 synthesis to ensure proper growth and glucose homeostasis.

### 4.3. Non-Coding RNAs in IGF and IGFR Regulation

Along with RBPs, non-coding RNAs including miRNAs and lncRNAs have emerged pivotal regulators of gene expression [18,28,126,127]. In this section we summarize miRNAs and lncRNAs that regulate the expression of IGF and IGFR. Unlike *insulin* and *IR* mRNAs, IGF and IGFR are known to be regulated by several miRNAs as discussed below.

Several studies identified a number of miRNAs affecting IGF-1. Among them, miR-29 was shown to target 3′ UTR of *IGF-1* mRNA to suppress IGF-1 expression. In hepatic satellite cells miR-29 levels decreased during myofibroblastic transition resulting in increased IGF-1 expression [128]. In mouse neonatal cardiomyocyte, miR-1 was found to suppress IGF-1 expression. While insulin treatment suppressed miR-1 levels and thus enhanced IGF-1 expression in cardiac and skeletal muscle, glucose stimulation increased miR-1 expression leading to low abundance of IGF-1 in the rat cardiomyocyte cell line H9C2 [22,129,130]. miR-206 and miR-320 were also found to target *IGF-1* mRNA in rat myoblast or myocardial microvascular endothelial cells of the type 2 diabetic Goto-Kakizaki rats [130,131]. The 3′ UTR of *IGF-2* mRNA was found to be targeted by miR-125b in C2C12 cells. During myoblast differentiation or muscle regeneration, miR-125b levels decreased leading to enhanced IGF-2 expression [23]

IGF-1R expression is regulated by several miRNAs including miR-7, miR-100, miR-139, miR-145, miR-223, miR-375, miR-378, miR-470, miR-669b and miR-681. In tongue squamous cell carcinoma cells, pre-miR-7 overexpression reduced IGF-1R protein level causing apoptosis and inhibition of proliferation [132]. It has been reported that miR-7 influences cancer cell migration and invasion by targeting IGF-1R [133]. During C2C12 differentiation, miR-133 abundance is elevated to suppress the expression of IGF-1R [134]. In venous smooth muscle cells miR-223 and miR-153 target *IGF-IR* mRNA and suppress IGF-IR expression [135]. In cardiomyocytes, miR-378 is upregulated during differentiation and targets *IGF-1R* mRNA to reduce its expression [136]. Additionally, *IGF-1R* 3′ UTR contains two potential sites for miR-675-3p, which is generated from lncRNA H19 processing. miR-675 has been found to suppress *IGF-1R* mRNA translation limiting placental growth before birth [26].

The influence of miRNAs on the expression of insulin system mRNAs is not only limited to muscle cells and differentiation but also extends to cancer cells since IGF and IGFR are crucial for tumor growth. For instance, in colorectal cancer (CRC), downregulation of miR-139 is associated with increased levels of its target IGF-1R and disease progression [137]. In Childhood adrenocortical tumors, miR-100 is downregulated allowing increased expression of its targets including *IGF-1R* and *mTOR* [24]. In colon cancer cell line HCT116, miR-145 negatively regulates IGF-1R expression [138]. In T-cell acute lymphoblastic leukemia, overexpression of miR-223 was found to reduce IGF-1R levels [139]. In oesophageal squamous cell carcinoma, miR-375 was found to negatively regulate IGF-1R expression to inhibit cell migration, colony formation, and tumor size [140]. In melanoma, miR-376a and miR-376c are significantly downregulated which may contribute into high expression of IGF-1R [141]. miR-470, miR-669b, and miR-681 are upregulated in GH-deficient mice leading to reduced expression of IGF-1R [142]. Recently it has been reported that miR-150* and miR-630 destabilize *IGF-1R* mRNA which leads growth arrest and apoptosis in pancreatic cancer cells [143].

Long non-coding RNAs (lncRNAs) have emerged as regulators of gene expression at multiple levels including chromatin remodeling, transcription, posttranscription, and protein metabolism [20,126]. Posttranscriptionally, lncRNAs are involved in splicing, mRNA turnover and translation and additionally they act as miRNA decoy [20]. Transcriptome analysis of human pancreatic islets β cells revealed tissue specific lncRNAs that are dynamically regulated and altered in type 2 diabetes. Downregulation of a β-cell-specific lncRNA, HI-LNC25, reduced the mRNA levels of GLIS3 (GLIS family zinc finger 3), which is involved in the development of pancreatic β-cells and associated diabetes [25]. In the insulin system two lncRNAs are known to regulate IGF-1R expression indirectly and directly. LncRNA H19 indirectly regulates IGF-1R expression since it is processed to miR-675 which represses translation of *IGF-1R* mRNA in placenta to limit its growth before birth [26]. Recently, the lncRNA Airn was found to directly regulate *IGF-2R* through transcription [27,144]. These findings indicate that lncRNAs may play crucial roles in pancreatic β-cell programming and diabetes pathophysiology. Future studies are warranted to identify potential lncRNAs that may directly regulate the insulin system at the posttranscriptional levels.

## 5. Concluding Remarks and Perspectives

**Figure 1:**
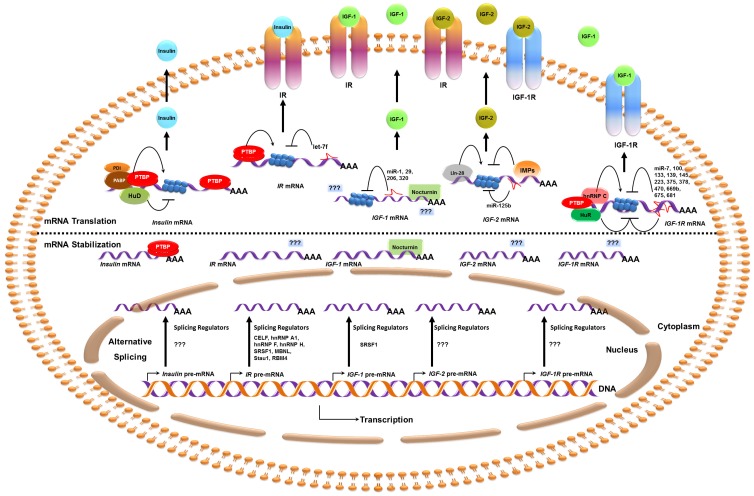
Posttranscriptional regulators of insulin system (ligands and receptors). Alternative splicing (bottom) is shown in the nucleus and the question marks indicate unknown regulators. PTBP and nocturnin promote the stabilization of *insulin* and *IGF-1* mRNAs respectively. Question marks indicate unknown factors that may regulate mRNA stability. While HuD suppresses, PDI, PABP and PTBP promote *insulin* mRNA translation (Upper part of the schematic). PTBP promotes *IR* mRNA translation which is suppressed by let-7. miR-1, 29, 206, and 320 suppress *IGF-1* mRNA translation, however *trans-*acting factors that regulate mRNA translation are not known. Lin-28 enhances *IGF-2* mRNA translation while IMPs and miR-125b suppress it. HuR suppresses, while hnRNPC and PTBP enhance *IGF-1R* mRNA translation. Several miRNAs are known to target the 3′ UTR to suppress *IGF-1R* mRNA translation. Finally, insulin ligands are secreted where they bind their receptors to transduce signaling events (top of the schematic).

Diabetes is one of the most common diseases in the United States and is also associated with other diseases such as cancer [2,3]. Diabetes could be an outcome of deregulated expression of the insulin system including insulin, IR, IGF, and IGFR [86]. Diabetes is mostly caused by reduced insulin synthesis from the pancreatic β-cells or reduced glucose responsiveness of the body which results from reduced or altered expression of IR or IGFR or both. The synthesis and secretion of insulin in pancreas is mostly controlled by glucose, which enhances insulin secretion followed by upregulation of insulin translation without affecting transcription in the early time [4]. This response is usually quick and effective in healthy individuals but can be altered due to genetic or acquired factors causing impairment in glucose metabolism and high susceptibility to develop diabetes. Additionally, altered levels of IGF and IGFR can enhance abnormal growth, obesity, diabetes and cancer [86]. The expression of these insulin family genes and their receptors are regulated at the posttranscriptional levels by several *trans-*acting factors. These factors regulate major steps including alternative splicing and mRNA stability and translation (Figure 1 and Table 1).

Alternative splicing is a major contributor in protein isoform diversity. Although splice variants for *insulin* mRNA are well characterized the alternative splicing regulators are not known. Due to the association of insulin levels with pathological conditions, it is important to identify the regulators of insulin alternative splicing which may represent a new avenue for treatments or disease prevention. Except for *IR* exon 11 alternative splicing, the mechanism of splice variant generation of the rest of the genes remains elusive. The discovery of new regulators in combination with the known ones will give further insight into the posttranscriptional regulation of the insulin family ligands and their receptors. In addition, since alternative splicing contributes to length variability in the UTRs of the transcripts, it is possible that alternative splicing is orchestrating with translation efficiency and stability to regulate their expression. Similarly, splice variants of IGF and IGFR are well studied, but splicing regulatory RNA-binding proteins are largely unknown. Uncovering these regulators is important since IGF and IGFR are not only involved in diabetes but also in cancer progression [86] and they could be targeted in therapeutic approaches. Moreover, several alternative splicing factors have been implicated in other aspects of mRNA regulation such as mRNA export, localization and translation and/or stability directly such as the SR proteins, CELF and MBNL [62,69,145]. The discovery of new alternative splicing factors will likely improve our understanding Insulin system regulation.

TTR-RBPs forming ribonucleoprotein complexes with *insulin* mRNA could be influenced by signaling events upon glucose treatments. For example, dissociation of HuD from *insulin* mRNA in glucose treated β-cells could be mediated by modifications such as protein phosphorylation. Indeed, HuD was found to be phosphorylated by PKC in neuronal cells [146]. However, it is important to investigate if kinases are involved in HuD-mediated effect on *insulin* mRNA translation. HuD is regulated by miR-375 in neuronal cells which suppresses dendrite extension by lowering HuD levels [42]. Although pancreatic β-cells express HuD, the levels of HuD in neuronal cells remain much higher likely due to different levels of miR-375. It has been reported that miR-375 is down regulated in senescent cells [147]. This may imply that miR-375 may indirectly regulate insulin through HuD, which suggests a possible interplay of HuD and miR-375 in insulin biosynthesis in old age. Additionally, it is not known if *insulin* mRNA is regulated by non-coding RNAs including lncRNAs and miRNAs. Although bioinformatic predictions indicated several miRNAs can potentially target *insulin* mRNA, experimental evidence, and pathological relevance remain to be investigated.

*IR* mRNA translation is regulated by PTBP1 and PTBP2 through binding to the 5′ UTR. However, it is not known if other TTR-RBPs bind and regulate *IR* mRNA. *IR* mRNA was found to be more stable in IR-rich HepG2 cells than IR-sparse MCF-7 cells. Under stress conditions such as growth arrest, *IR* mRNA half-life increases in both cell lines indicating that *IR* mRNA stabilization is a key factor in the regulation of IR expression [148]. It is also important to note that the 3′ UTR of *IR* mRNA contains several AU-rich sequences which could be responsible for mRNA stabilization or degradation [149]. These findings strongly suggest that *IR* mRNA may form ribonucleoprotein complexes with other RBPs such as HuD, HuR or hnRNP D to regulate *IR* mRNA half-life [127]. In fact, mapping HuR binding sites using PAR-CLIP revealed several hits in the 3′ UTR of *IR* mRNA [150]. However, the influence of HuR on *IR* mRNA remains to be investigated. Additionally, other PAR-CLIP studies indicated that Lin-28 and fragile X mental retardation protein (FMRP) can bind the coding region (CR) and the 3′ UTR of *IR* mRNA [151,152]. These findings strongly suggest possible roles for Lin-28, FMRP, and possibly other TTR-RBPs in the regulation of IR expression. Let-7 miRNA regulates IR expression in addition to IR substrate 2 [21]. This could represent a clinical opportunity to utilize miRNA-based therapy for IR-associated diseases. Moreover, miRNAs are known to target several genes and one gene can be targeted by several miRNAs. Indeed, miRNA prediction algorithms indicate that *IR* mRNA can be potentially targeted by several miRNAs. Thus, it is important to investigate if other miRNAs could regulate IR expression and provide pathological and clinical relevance.

Nocturnin binds to the 3′ UTR of *IGF-1* mRNA to regulate its stability [108]. It is not known however if it is the only regulator of *IGF-1* mRNA or other TTR-RBPs could be involved. mRNAs encoding for IGF and IGFR proteins contain relatively long 3′ UTR with sequences that are suitable for binding to other RBPs. Mapping HuR binding using PAR-CLIP indeed revealed several hits in *IGF-1R* and *IGF-2R* mRNAs [150]. This confirms HuR binding to *IFG-IR* mRNA to suppress its translation [15]. However, the influence of HuR on *IGF-2R* mRNA remains to be investigated.

Lin-28 enhances *IGF-2* mRNA translation through binding to the 5′ UTR [16]. However, CLIP data indicated that Lin-28 can bind the CR and the 3′ UTR of *IGF-1R* mRNA and introns in the pre-mRNA, while it may bind to intron and coding region of *IGF-2R* mRNA [151]. Similarly, FMRP may also bind *IGF-1R* mRNA in the CR and the 3′ UTR, with one binding site in the pre-mRNA [152]. These data suggest that Lin-28 and FMRP could be involved in posttranscriptional regulation of *IGF-1R* and *IGF-2R* mRNA. Future studies will confirm and reveal the importance of these RBPs in the regulation of the insulin system and their implications in glucose homeostasis.

LncRNAs have emerged as posttranscriptional regulators of gene expression together with miRNAs and RBPs [20]. Although lncRNAs (HI-LNC25, H19, and Arin) have been linked to the insulin system, it is not known whether lncRNAs have direct posttranscriptional influence on insulin system. Furthermore, regulatory lncRNAs may be differentially expressed in diabetic conditions or even altered during disease progression. Future studies will reveal whether lncRNAs are directly associated with the insulin system either through lncRNA-mRNA or lncRNA-protein interaction.

In closing, although a great deal of knowledge is already available; several questions remain unanswered (Figure 1 and Table 1). Understanding posttranscriptional events will help improve our understanding of the insulin system regulation and may also provide additional avenues for treatments and disease prevention.

## Figures and Tables

**Table 1:** Posttranscriptional regulators of insulin family ligands and their receptors.

Target	Regulators	Target Region(s)	Functions	Condition/Disease	Reference
Insulin	PABP	5′ UTR	Translation activation	High glucose	[39]
	PDI	5′ UTR	Translation activation	High glucose	[39]
	HuD	5′ UTR	Translation repression	Low glucose	[43]
	PTBP	3′ UTR	mRNA stabilization	Hypoxia and High glucose	[13,46]
	PTBP	5′ UTR	IRES translation activation	Nitrosative stress	[47]

IR	CELF1	Intron 10, Exon 11	Exon 11 skipping	Muscular Dystrophy	[63,66]
	SRp20	Exon 11	Exon 11 inclusion	Basal	[63]
	SRSF1	Exon 11	Exon 11 inclusion	Basal	[63,68]
	hnRNP H	Intron 10	Exon 11 skipping	Muscular Dystrophy	[66]
	hnRNP A1	Intron 10, Exon 11	Exon 11 skipping	Muscular Dystrophy	[68]
	hnRNP F	Intron 10	Exon 11 inclusion	Basal	[68]
	MBNL	Intron 10, 11 and exon 11	Exon 11 inclusion	Basal	[63,66,71]
	RBM4	NA	Exon 11 inclusion	High glucose	[76]
	PTBP1, PTBP2	5′ UTR	IRES translation activation	Insulin stimulus	[79]
	LIN-28	CR and 3′ UTR	NA	Basal	[151]
	FMRP	CR and 3′ UTR	NA	Basal	[152]
	let-7f	3′ UTR	Translation Repression *	Diabetes	[21]

IGF-1	SRSF1	Exon 5	Exon 5 inclusion	Growth hormone treatment	[103]
	Nocturnin	3′ UTR	mRNA decay	Circadian rhythm	[108]
	miR-29	3′ UTR	mRNA decay	Fibrosis	[128]
	miR-1	3′ UTR	Translation repression	Muscle differentiation	[22]
	miR-1	3′ UTR	Translation repression	Apoptosis	[129,130]
	miR-206	3′ UTR	Translation repression	Apoptosis	[130]
	miR-320	3′ UTR	Translation repression *	Angiogenesis	[131]

IGF-2	Lin28	NA	Translation activation	Skeletal myogenesis	[16]
	IMP1, IMP2, IMP3	5′ UTR (L3)	Translation repression	Embryogenesis	[121]
	IMP3	5′ UTR (L3 and L4)	Translation activation	Cell proliferation	[122]
	IMP2	5′ UTR (L3)	IRES translation activation	Embryogenesis	[17]
	miR-125b	3′ UTR	mRNA decay	Muscle regeneration	[23]

IGF-1R	HuR	5′ UTR	Translation repression	Amino acid deprivation	[15]
	HuR	5′ UTR	IRES translation repression	Amino acid deprivation	[15]
	hnRNP C	5′ UTR	IRES translation activation	Metaphase block	[115]
	PTBP	5′ UTR	IRES translation activation	Basal	[119]
	LIN-28	Intron, CR and 3′ UTR	NA	Basal	[151]
	FMRP	Intron, CR and 3′ UTR	NA	Basal	[152]
	miR-7	3′ UTR	mRNA decay	Apoptosis	[132]
	miR-7	3′ UTR	Translation repression	Metastasis	[133]
	miR-100	3′ UTR	Translation repression *	Adrenocortical tumors	[24]
	miR-133	3′ UTR	Translation repression	Muscle differentiation	[134]
	miR-145	3′ UTR	Translation repression	Colon cancer	[138]
	miR-223	3′ UTR	Translation repression	Muscle differentiation	[135]
	miR-223	3′ UTR	Translation repression	Lukemia	[139]
	miR-375	3′ UTR	mRNA decay	Cancer	[140]
	miR-378	3′ UTR	Translation repression	Cardiomyocyte survival	[136]
	miR-675-3p	3′ UTR	mRNA decay	Placental growth	[26]
	miR-139	3′ UTR	mRNA decay	Colorectal cancer	[137]
	miR-376a and 376c	3′ UTR	mRNA decay	Melanoma	[141]
	miR-470, 669b and 681	3′ UTR	Translation repression *	GH-deficiency	[142]
	miR-150* and 630	3′ UTR	mRNA decay	Growth arrest and apoptosis	[143]

IGF-2R	HuR	Intron and 3′ UTR	NA	Basal	[150]
	LIN-28	Intron and CR	NA	Basal	[151]
	FMRP	CR	NA	Basal	[152]

* Likely regulatory mechanism.

## Abbreviations

CR: coding region
CELF: CUG-binding protein and Elav-like family
ELAV: embryonic lethal abnormal vision
hnRNP: heterogeneous nuclear ribonucleoprotein
IGF: insulin-like growth factor
IGFBP: IGF binding protein
IGF-1R: IGF-1 receptor
IIS: insulin and IGF signaling
IMP: IGF-2 mRNA binding proteins
IR: insulin receptor
lncRNA: Long non-coding RNA
MBNL: muscle blind-like
miR: microRNA
PABP: poly(A)-binding protein
PTBP: polypyrimidine tract-binding protein
RBM4: RNA-Binding Motif Protein 4
RBP: RNA-binding protein
SRSF1: serine/arginine-rich splicing factor 1
TTR-RBP: turnover and translation regulator RNA-binding proteins
UTR: untranslated region
